# Writing, Proofreading and Editing in Information Theory

**DOI:** 10.3390/e20050368

**Published:** 2018-05-15

**Authors:** J. Ricardo Arias-Gonzalez

**Affiliations:** 1 Instituto Madrileño de Estudios Avanzados en Nanociencia, C/Faraday 9, Cantoblanco, 28049 Madrid, Spain; ricardo.arias@imdea.org; Tel.: +34-91-299-8860; 2 CNB-CSIC-IMDEA Nanociencia Associated Unit “Unidad de Nanobiotecnología”, Cantoblanco, 28049 Madrid, Spain

**Keywords:** thermodynamics, stochastic, non-Markovian, memory, proofreading, editing, replication, transcription, translation

## Abstract

Information is a physical entity amenable to be described by an abstract theory. The concepts associated with the creation and post-processing of the information have not, however, been mathematically established, despite being broadly used in many fields of knowledge. Here, inspired by how information is managed in biomolecular systems, we introduce writing, entailing any bit string generation, and revision, as comprising proofreading and editing, in information chains. Our formalism expands the thermodynamic analysis of stochastic chains made up of material subunits to abstract strings of symbols. We introduce a non-Markovian treatment of operational rules over the symbols of the chain that parallels the physical interactions responsible for memory effects in material chains. Our theory underlies any communication system, ranging from human languages and computer science to gene evolution.

## 1. Introduction

Many concepts related to the treatment of information have ordinarily been used for centuries, but only rigorously defined within the context of information theory by the end of the 1940s [1,2]. Examples are information content, as quantified by the entropy, or communication load, as characterized by the channel capacity. In the same way, error is normally used as a binary concept that refers to either a correct or an incorrect transfer of a symbol. Writing, including the creation of original text in human languages or the generation of genes in biomolecular systems, transcription or translation of information, as well as any kind of evolution of an initial information chain, however, cannot be simply assessed as correct or wrong because many strings of characters may carry similar information. At the level of a single subunit, sometimes, the replacement of a particular symbol, as characterized by the value of a random variable, may be indifferent or simply may not alter the content or meaning of an information chain.

The appropriate introduction of operational rules to construct bit strings is linked to the use of the concept of proofreading and, more importantly, to the establishment of conditions for useful editing [3]. These concepts appear together in biomolecular processes associated with information management [4,5,6]. Monomers, either nucleotides or amino acids, are chained according to a template sequence. A replicated DNA strand or an RNA transcript thus represents a sequence of symbols that code for proteins; a protein, in turn, comprises a translated gene, an information chain that entails structural and functional fates in a cell. Physical interactions have been evolutionarily adapted in these natural systems generating a direct correspondence between the thermodynamic stability of a biopolymer chain and the transfer of its information [7,8].

Biomolecular engines that process genetic information work under non-equilibrium conditions [9], but not in ensembles in the cell. These systems are one of the most important subjects of stochastic thermodynamics [10], a relatively new extension of classical thermodynamics that deals with small systems [11]. The relations between equilibrium thermodynamic potentials and non-equilibrium work or entropy production through fluctuation theorems [12,13] and the burst of single-molecule biophysics [14], in which individual protein dynamics are experimentally followed [15], demonstrate the importance of studying individual trajectories. In this regard, it is paradigmatic that writing, proofreading and editing of information involve the composition of individual strings of symbols, therefore making the statistical treatment in stochastic thermodynamics useful in this purely informational context [16,17].

We previously formulated a thermodynamic framework for physical systems that carry information in the presence of memory effects [3] with general non-Markovian reach [18]. In this paper, we expand this framework to general systems, both symbolic and physical, conveying information, that is to systems where information content is not determined by thermodynamic laws, but able to respond to ad hoc, imposed operational rules, as is the case for human languages. We then express conditions for both effective proofreading and editing. In the second part, we apply our formalism to the process of copying information. We adapt a toy model that was formulated in the context of DNA replication and transcription and test the universal conditions for effective revision. We end with concluding remarks on our theory, including an outlook toward its alternative exploitation.

## 2. Analysis

The probability distribution, $\text{Pr}\left\{X=x\right\}=p\left(x\right)$, of a random variable *X* with alphabet X can be written as an exponential of an arbitrary real function, namely, $p\left(x\right)∼a^{f\left(x\right)}$, with a>1. For convenience, and without loss of generality, we will introduce probability distributions as:

(1) $$p\left(x\right)=\frac{1}{Q}a^{\frac{C\left(x\right)}{R}},$$

where $C\left(x\right)$ is a real function of the random variable *X* and R>0 is a parameter that modulates the weight of function *C* in the exponent. *Q* is a normalization factor, as determined from $Q=\sum_{x\in X}a^{\frac{C\left(x\right)}{R}}$. Function *C* is recovered from the probability distribution by taking the logarithm to base *a*, namely, $C\left(x\right)=R\log_{a}\left[Qp\left(x\right)\right]$, and therefore, $C\left(x\right)$ can be considered a transform of the probability distribution $p\left(x\right)$ that maps the image interval $\left[0,1\right]$ to $\left(-\infty ,R\log_{a}Q\right]$.

We next explain the convenience of expressing probabilities as in Equation (1). In particular, we provide meaning to function *C* in the context of a string of symbols, as characterized by a directional, stochastic chain with memory [19]. Let $\nu =\left\{x_{1},x_{2},\ldots ,x_{i},\ldots ,x_{n-1},x_{n}\right\}$ be a stochastic chain arranged 1→n (say forward or from left to right) by a linear sequence of symbols and/or physical objects, $x_{i}$, as values of random variables $X_{i}$. We will extend the concept of energy to general linear, stochastic chains, so that it can be useful in information theory.

**Definition** (Correctness) *Let ν be a stochastic sequence of symbolic, random variables $X_{i}$, i=1,…,n. We associate with each $X_{i}$ a real function called the* correctness*, $C_{i}$, following Equation (1), which, within ν, depends on both the symbol $x_{i}$ and the sequence of previous symbols $x_{i-1},\ldots ,x_{1}$, as $C_{i}\equiv C\left(x_{i};x_{i-1},\ldots ,x_{1}\right)$. We say that $C(x_{i};x_{i-1},\ldots ,x_{1})$ is the correctness of symbol $x_{i}$ provided that the previous symbols are $x_{i-1},\ldots ,x_{1}$. The correctness $C_{i}$ will be assigned to symbol $x_{i}$ on the basis of its precision within the chain ν. Specifically, the more correct symbol $x_{i}$, the higher its correctness $C_{i}$; the more erroneous $x_{i}$, the lower $C_{i}$.*

Since the probability of a chain can be written as [20]:

(2) $$p_{\nu }\equiv \text{Pr}\left\{X_{1}=x_{1},\ldots ,X_{n}=x_{n}\right\}=p\left(x\right)=p\left(x_{1},\ldots ,x_{n}\right)=p\left(x_{1}\right)p\left(x_{2}|x_{1}\right)\cdots p\left(x_{n}|x_{n-1},\ldots ,x_{1}\right),$$

it is possible to define the total correctness of a sequence, $C_{\nu }$, as the sum over all the correctness of its symbols:

(3) $$C_{\nu }\equiv C\left(x\right)=C\left(x_{1},\ldots ,x_{n}\right)=\sum_{i=1}^{n}C_{i}\left(x_{i};x_{i-1},\ldots ,x_{1}\right).$$

The correctness is therefore an additive property of the subunits within the chain.

The interactions, which determine a memory, between subunits in a material chain, as for example in polynucleotide biopolymers [19], may be naturally imposed by the physics of the systems [3]. In these chains, the energy plays the role of the correctness (C→−E), and parameter *R* is associated with the thermal energy level, namely R→kT, where *k* is the Boltzmann constant and *T* the temperature. This suggests the modeling of function *C* as a strategy to introduce ad hoc the operational rules in purely symbolic systems, like in human communication. In this case, each individual contributes differently to the correctness since his/her own capacity to assimilate the operational rules of a specific language entails different protocols for their application, as reflected in the probabilities of Equation (1). The action of a human being in text composition is analogous to those of the DNA and RNA polymerases in replication and transcription, respectively. These nanomachines introduce non-equilibrium protocols with energy dissipation, which adds to the hybridization free energies of the resulting nucleic acids base-pairs [3,19].

Note that replacing *C* by $C+C_{0}$, $C_{0}$ being a constant, affects the correctness, but not the probabilities. This arbitrariness is also present in Hamiltonian systems, where the reference for potential energies is not universal.

The individual outcomes of a multivariate stochastic process with memory or its time-evolution cannot be rated just as erroneous or correct because several degrees of accuracy may be possible. The correctness function allows extending the concepts of error and certainty (herein used as the opposite of error). Specifically, a stochastic sequence, ν, will be said to be erroneous when its associated correctness, $C_{\nu }$, is low. The lower $C_{\nu }$, the more erroneous ν. On the contrary, a chain ν will be said to be certain when its associated correctness, $C_{\nu }$, is high. The higher $C_{\nu }$, the more correct ν.

The fact that $C_{\nu }$ is low or high is then the result of a balance between the number of errors (wrong $x_{i}$) and certainties (correct $x_{i}$) and their impact, as measured by the individual correctness, $C_{i}$. A high correctness is such that $C_{i}/R\gg 1$, and a low correctness, $C_{i}/R\ll 1$. In any case, it is necessary that $|C_{i}/R|\gg 1$ for the concepts of both certainty and error to make sense. In the absence of randomness, which can be studied in the limit of very low *R*, the above formalism leads naturally to what is expected for probabilities, namely that $p_{certain}=1$ and $p_{error}=0$.

The probability of a particular configuration in directionally-constructed stochastic chains [19] can now be written as:

(4) $$p_{\nu }^{\left(w\right)}=\frac{a^{\frac{C_{\nu }}{R}}}{Q_{\nu }},\;\;\;\;\;\;\;Q_{\nu }\equiv \sum_{\nu^{\prime }=1}^{N}a^{\frac{C_{\nu^{\prime }\nu }}{R}},$$

where $\sum_{\nu^{\prime }=1}^{N}$ stands for $\sum_{x_{1}^{\prime },\ldots ,x_{n}^{\prime }\in X}$, $Q_{\nu }$ is the sequence-dependent normalization, *N* the number of sequences and $C_{\nu^{\prime }\nu }$ the two-sequence correctness defined as [19]:

(5) $$C_{\nu^{\prime }\nu }\equiv \sum_{i=1}^{n}C\left(x_{i}^{\prime };x_{i-1},\ldots ,x_{1}\right),$$

with $C_{\nu \nu }=C_{\nu }$. Such chain-construction statistical treatment was shown to imply an irreversibility, which is inherent to the system as assessed by practical writing constraints (for example, when using a typewriting machine).

In contrast, if we do not restrict how to access each final configuration [3,19], the probability of a certain sequence reads:

(6) $$p_{\nu }^{\left(r\right)}=\frac{a^{\frac{C_{\nu }}{R}}}{Q},\;\;\;\;\;\;\;Q=\sum_{\nu =1}^{N}a^{\frac{C_{\nu }}{R}},$$

being *Q* the standard normalization. As with partition functions [3,19], the normalizations fulfill ${\langle 1/Q_{\nu }\rangle }_{r}=1/Q$ and ${\langle Q_{\nu }\rangle }_{w}=Q$, and the probabilities ${\langle p_{\nu }^{\left(w\right)}/p_{\nu }^{\left(r\right)}\rangle }_{r}=1$ and ${\langle p_{\nu }^{\left(r\right)}/p_{\nu }^{\left(w\right)}\rangle }_{w}=1$, where ${\langle \ldots \rangle }_{w}=\sum_{\nu =1}^{N}p_{\nu }^{\left(w\right)}\ldots$ and ${\langle \ldots \rangle }_{r}=\sum_{\nu =1}^{N}p_{\nu }^{\left(r\right)}\ldots$

The formalism given by Equation (6) involves a reversible process and therefore comprises the concept of revision, in which backward recognition of errors (proofreading) and their substitution by new objects (editing) take place [3,19] (as, for example, when using a word processor in a computer). Therefore, we will extend previous results, hence calling the process represented by Equation (4) writing and that represented by Equation (6) revision, thus justifying the use of superindices *w* and *r*, respectively.

We now introduce the edition potential, “*A*”, and the entropy, “*H*”, for single chains. Depending on whether we are writing or proofreading and editing, these functions read:

(7) $$\begin{array}{cc} & A_{\nu }^{\left(w\right)}\equiv R\log_{a}Q_{\nu },\;A_{\nu }^{\left(r\right)}\equiv R\log_{a}Q, \end{array}$$

(8) $$\begin{array}{cc} & H_{\nu }^{\left(w\right)}\equiv -\log_{a}p_{\nu }^{\left(w\right)},\;\;H_{\nu }^{\left(r\right)}\equiv -\log_{a}p_{\nu }^{\left(r\right)},\; \end{array}$$

fulfilling:

(9) $$\;A_{\nu }^{\left(w\right)}=C_{\nu }+RH_{\nu }^{\left(w\right)},\;A_{\nu }^{\left(r\right)}=C_{\nu }+RH_{\nu }^{\left(r\right)},$$

which is the energy conservation analogue. It is noted that the edition potential does not depend on a particular chain, ν, for the case of revision (Equation (7), right). Besides, the correctness of a particular chain, $C_{\nu }$, does not depend on how it has been constructed (whether with or without revision). Functions “*A*” and “*H*” in Equations (7) and (8) characterize the generation of information and the information content, respectively, of an individual symbolic chain under the rules modeled through function *C*.

Respective functionals can be constructed by taking ensemble-averages:

(10) $$\begin{array}{cc} & C^{\left(w\right)}\equiv {\langle C_{\nu }\rangle }_{w},\;\;\;\;\;\;\;C^{\left(r\right)}\equiv {\langle C_{\nu }\rangle }_{r};\; \end{array}$$

(11) $$\begin{array}{cc} & A^{\left(w\right)}\equiv R{\langle \log_{a}Q_{\nu }\rangle }_{w},\;A^{\left(r\right)}\equiv R\log_{a}Q;\; \end{array}$$

(12) $$\begin{array}{cc} & H^{\left(w\right)}=-{\langle \log_{a}p_{\nu }^{\left(w\right)}\rangle }_{w},\;\;\;H^{\left(r\right)}=-{\langle \log_{a}p_{\nu }^{\left(r\right)}\rangle }_{r}; \end{array}$$

where $R\log_{a}Q=R\langle \log_{a}Q\rangle$ in Equation (11), right. Equations for writing (left) and revision (right) are formally analogous. Importantly, we recover the definition of entropy in information theory for a=2 [2]. These functionals characterize the average correctness, Equation (10), the generation of information, Equation (11), and information content, Equation (12), of chains constructed under the rules modeled through function *C* in the absence (superindex *w*) or presence (*r*) of revision.

The analogue of the energy conservation for ensemble-average phenomena yields:

(13) $$\;A^{\left(w\right)}=C^{\left(w\right)}+RH^{\left(w\right)},\;\;\;\;A^{\left(r\right)}=C^{\left(r\right)}+RH^{\left(r\right)},$$

which arise by formally taking expected values in Equations (9). The demonstrations of Equations (9)–(13) are straightforward from their thermodynamic analogues [3]. The following relations can also be deduced from their thermodynamic analogues [3]:

(14) $$\begin{array}{cc} & C^{\left(w\right)}={\langle \frac{\partial }{\partial B}\log_{a}Q_{\nu }\rangle }_{w},\;\;C^{\left(r\right)}=\frac{\partial }{\partial B}\log_{a}Q; \end{array}$$

(15) $$\begin{array}{cc} & H^{\left(w\right)}={\langle \frac{\partial }{\partial R}A_{\nu }^{\left(w\right)}\rangle }_{w},\;\;H^{\left(r\right)}=\frac{\partial }{\partial R}A^{\left(r\right)}; \end{array}$$

where B≡1/R. Since $\frac{\partial }{\partial B}\log_{a}Q=\langle \frac{\partial }{\partial B}\log_{a}Q\rangle$ and $\frac{\partial }{\partial R}A=\langle \frac{\partial }{\partial R}A\rangle$, expressions on the left (writing), again, are formally analogous to those on the right (revision) in Equations (14) and (15).

The following inequalities hold [3]:

(16) $$\begin{array}{cc} & A^{\left(r\right)}-A^{\left(w\right)}\;\geq \;0, \end{array}$$

(17) $$\begin{array}{cc} & H^{\left(r\right)}-H^{\left(w\right)}\geq -\frac{1}{R}\left(C^{\left(r\right)}-C^{\left(w\right)}\right). \end{array}$$

In addition, it is straightforward to see that $A^{\left(r\right)}\geq C^{\left(r\right)}$ and that $A^{\left(w\right)}\geq C^{\left(w\right)}$, which together with Inequality (16), make $A^{\left(r\right)}\geq C^{\left(w\right)}$.

These expressions allow extending the conditions for effective revision that were found for thermodynamic systems carrying information [3]:

**Condition 1** (Effective editing necessary and sufficient condition). (18) $$\begin{array}{ccc} Effective\;editing & \Leftrightarrow & H^{\left(r\right)}\leq H^{\left(w\right)}, \end{array}$$

(19) $$\begin{array}{ccc} Ineffective\;editing & \Leftrightarrow & H^{\left(r\right)}>H^{\left(w\right)}. \end{array}$$

**Condition 2** (Effective proofreading necessary and sufficient condition). (20) $$\begin{array}{ccc} Effective\;proofreading & \Leftrightarrow & C^{\left(r\right)}\geq C^{\left(w\right)}, \end{array}$$

(21) $$\begin{array}{ccc} Ineffective\;proofreading & \Leftrightarrow & C^{\left(r\right)}<C^{\left(w\right)}. \end{array}$$

The equalities take place when there exists no memory (see the independence limit theorem [19]). Note that the introduction of an arbitrary constant in the definition of the correctness (say $C_{0}$; see above) does not affect the formulation of these conditions.

**Corollary 1** (Effective editing necessary condition and effective proofreading sufficient condition). (22) Effectiveediting⇒Effectiveproofreading,

**Corollary 2** (Ineffective editing sufficient condition and ineffective proofreading necessary condition). (23) Ineffectiveproofreading⇒Ineffectiveediting

Corollaries 1 and 2 are consequences of Conditions 1 and 2 after the use of Equation (17).

## 3. Application: Copying

We envision a system, either natural or artificial, that computes information by assembling characters in the same way as a so-called Turing machine and according to adopted rules, as characterized by the correctness. The characters are symbolic elements from an alphabet set, with the independence of the fact that they might have material reality, like atoms or molecules in a physical realization of the system. The alphabet, X, is conformed by a number |X| of characters correlated with symbolic operational rules, with independence of whether they might be implemented through existing interactions in a physical realization of the system. In the case of biomolecular systems in cells, intrinsic physical interactions have been accommodated to encode information through evolution. Under the action of protein motors, this information is transferred and subjected to mutations. In the case of human language, symbolic rules have been evolved throughout history, with persons interpreting them and managing information.

In the following, we model the copy of information in a binary-alphabet classical system (|X|=2, a=2). To this end, we adapt a toy model that was previously developed in the context of DNA replication and transcription [3,19,21]. We propose that at each memory position, the introduction of a bit value (0/1) adds a contribution ±ξ (ξ, a real, positive parameter, which we will name stability contrast) to the correctness of the string and that this contribution is affected through the coupling with the previous bit values (memory), as modeled by a power law (see Supplementary Materials). The coupling strength is controlled by parameter α in the exponent, which increases for decreasing memory effects. Memory effects specifically introduce either a positive or negative feedback, thus increasing or decreasing the amount ±ξ. For reference, when the feedback is positive or when it is negative, but the memory effects are not too strong, a bit value that contributes with +ξ to the correctness is assimilated as a correct incorporation and a value with −ξ as an error.

**Figure 1:**
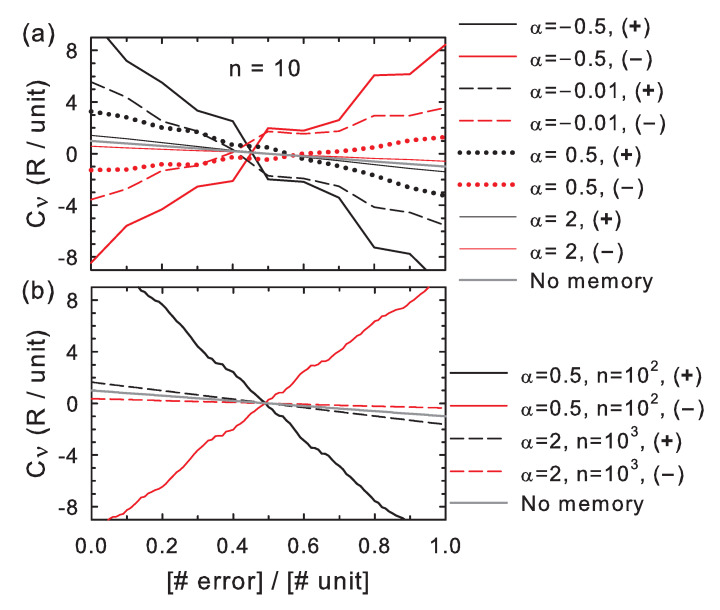
Correctness as a function of the number of errors. Both axes in the graphs are scaled to the number of memory units (*n*). The stability contrast between correct and wrong insertions is fixed to ξ=R. The memory is modeled by a power law correlation between each incorporated symbol and the previous ones with exponent parameter α (Supplementary Materials). The curves show trends for individual realizations of a copying process for several α, subjected to either positive or negative feedback ((+) or (−) in the legends), for n=10 (**a**), and n=100 or 1000 (**b**).

The correctness in individual bit strings decreases with the number of errors for positive feedback and for negative feedback under weak memory effects (high α), as shown in Figure 1, which is expected for a rationally-designed copying system. When the feedback is negative and memory effects are sufficiently strong (low α), however, the correctness increases with the number of errors, which indicates that the correlations with the previous bit values reverse the effect of the a priori correct/incorrect bit values (+ξ/−ξ). In both positive and negative feedback cases, the correctness approaches the case of independent variables when memory effects become negligible (α→∞). The curves further reveal that the correctness increases/decreases at each memory position, *i*, in steps of size ξ swayed by the effect of the feedback from the previous bit values in the sequence.

**Figure 2:**
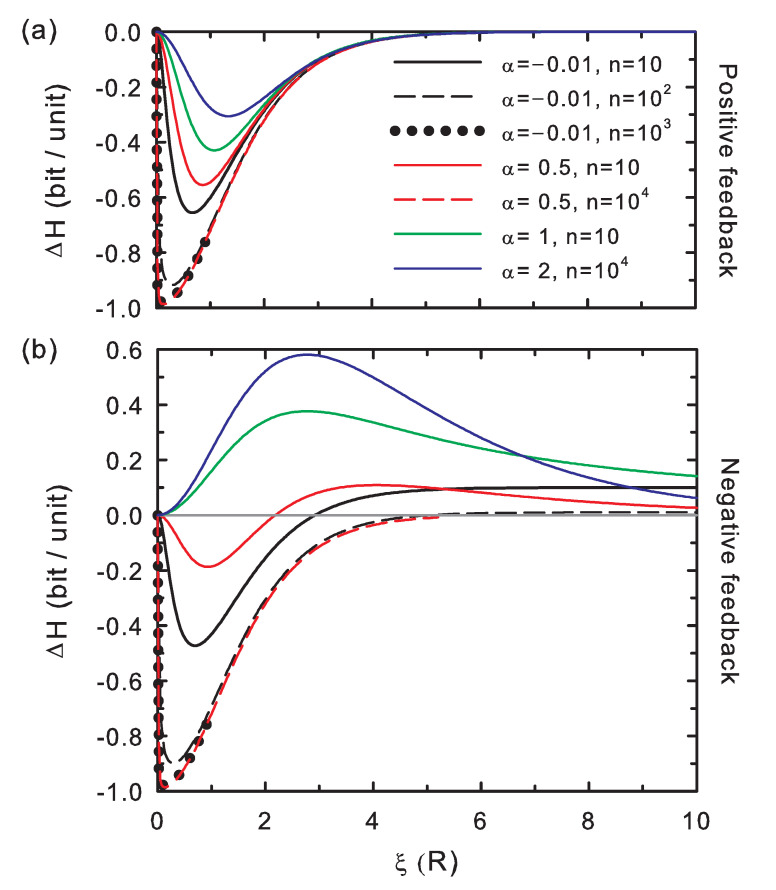
Entropy difference per memory unit as a function of the stability contrast between correct and wrong insertions. The memory is modeled by establishing a power law correlation between each incorporated symbol and the previous ones (Supplementary Materials). The curves show the behavior of $\Delta H=H^{\left(r\right)}-H^{\left(w\right)}$ for several chain lengths (*n*) and coupling strengths (α). (**a**) Positive feedback; (**b**) negative feedback.

The entropy difference scaled to the number of memory positions, as shown in Figure 2, ranges between [−1,+1], as expected for a binary alphabet. This potential difference exhibits negative values for positive feedback, implying that revision improves the information content of the bit string (Condition 1). In the case of negative feedback, ΔH becomes positive for weak memory effects, according to an ineffective revision (Condition 1). For strong negative feedback conditions (α≲1), sufficiently long strings and low values of the stability contrast, this potential becomes again negative. In this case, information is generated (Condition 1) by opposites: a bit value that contributes with +ξ to the correctness generates an overall negative contribution to the correctness due to the strong memory effects (and may be now assumed as an error) and that with −ξ an overall negative contribution (and may be now considered as a correct incorporation). The information generation in this scenario is, however, of less quality than in the positive-feedback scheme: this strongly-correlated information system with negative feedback rewards the introduction of the above termed a priori errors.

**Figure 3:**
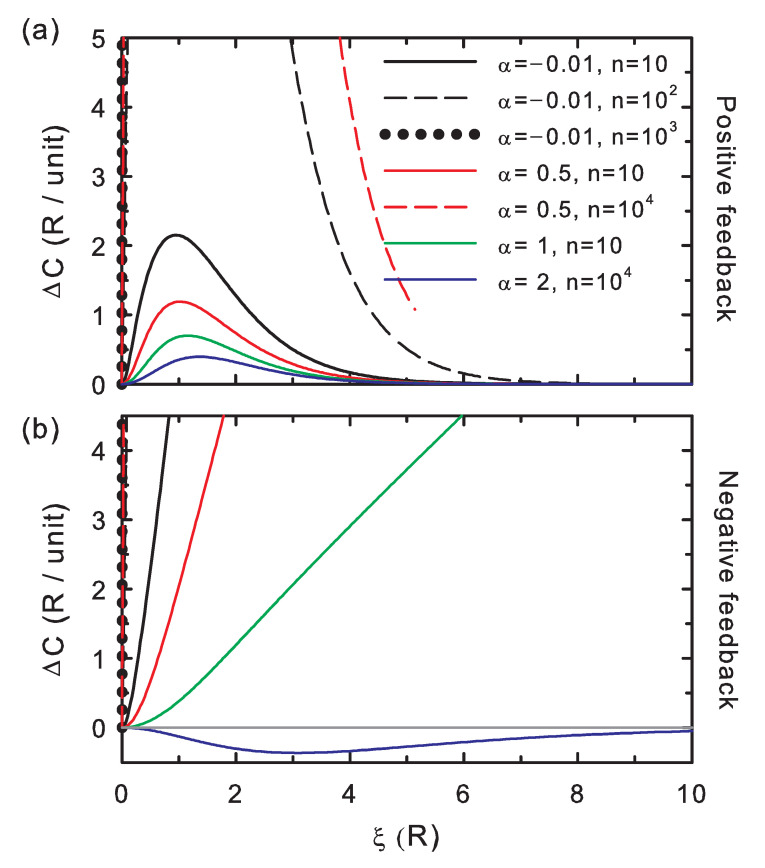
The same as in Figure 2 for the correctness potential difference per unit memory. (**a**) Positive feedback; (**b**) negative feedback.

The correctness potential difference scaled to the number of memory positions, (Figure 3) is positive for positive feedback, which indicates that the proofreading of the strings generated this way is effective (Condition 2). As explained above for Figure 2, the fidelity of the copy increases (all in all, complying with Corollary 1). For negative feedback, there are again two regimes: for weak memory effects (high α), the correctness difference becomes negative, which indicates that proofreading is ineffective (Condition 2) and makes the editing ineffective, as well (Corollary 2). In the strongly-correlated limit (low α) and negative feedback, the correctness becomes again positive. This is compatible with revision being effective and ineffective, which, as explained for Figure 2, depends on the string length and stability contrast regime. Information, as mentioned, is generated by a counter-balance effect of the memory. This is, again, coherent with the formulated revision Condition 1 and Corollary 1.

**Figure 4:**
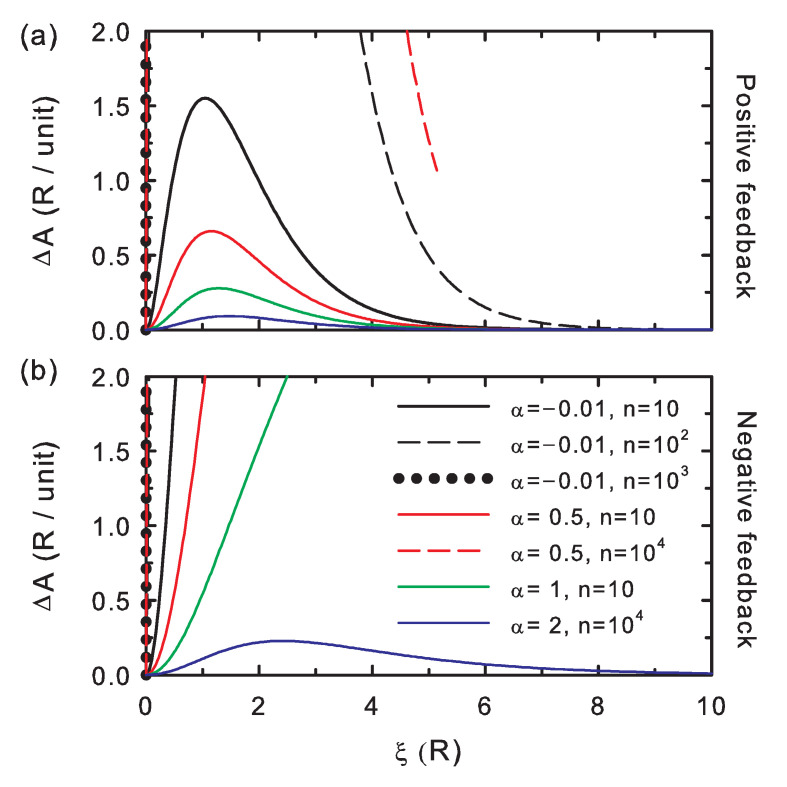
The same as in Figure 2 for the edition potential difference per unit memory. (**a**) Positive feedback; (**b**) negative feedback.

The edition potential difference (Figure 4) is always positive, as described by Equation (16); hence, it is also useful as a control check for the simulations. The larger its values, the more distinct the bit string after editing. The curves show that ΔA increases for increasing interaction strength (i.e., decreasing coupling parameter, α) and for increasing chain length (*n*).

These appear to be the general trends for the absolute value of the correctness potential difference: |ΔC| overall increases for both increasing interactions (decreasing α) and chain length (*n*), Figure 3. Both trends are overall followed by the absolute value of the entropy potential difference (Figure 2), although for the negative feedback regime, |ΔH| must be analyzed with care along the stability contrast coordinate, ξ, for α≈1, which separates the strong and weak coupling regimes.

## 4. Conclusions

We have shown that information strings can be studied in analogy with the Hamiltonian treatment of stochastic chains with memory. We have introduced the correctness, which parallels the concept of energy, and have formulated conditions for effective proofreading and editing. When considering thermodynamics in a physical realization of an information system, it is important to note that time is implicitly included in our formalism, either by conceiving of indefinitely long intervals or by addressing this coordinate in the evolution of the correctness function.

Revision, as introduced in this report, entails an equilibrium thermalization in the physical analogy, hence comprising an infinite number of proofreading and editing rounds. The problem of considering one or a finite number of finite-time revisions involves a non-equilibrium modeling analogue.

This work represents a round trip: from information theory to stochastic thermodynamics, non-Markovian dynamics and molecular biophysics, and back. This trip affords for the first time the introduction of writing, proofreading and editing in information theory, with a sufficient degree of conceptualization to be used in social and natural sciences, including the physics of stochastic systems and nanoscale engineering. Our formalism enables semantic roots to bit sequences, which are important in gene evolution analysis and in context-sensitive error correction algorithms for computational linguistics, currently based on statistical occurrences.

## Supplementary Materials

The supplementary materials are available online at http://www.mdpi.com/1099-4300/20/5/368/s1.
