# Supplementary material for: Writing, Proofreading and Editing in Information Theory
Source: Entropy (Basel). 2018 May 15;20(5):368. doi: 10.3390/e20050368 (PMC7512888; doi:10.3390/e20050368)
Supplement: Supplementary file 1 [file entropy-20-00368-s001.pdf]

Supplementary Materials

Writing, Proofreading and Editing in Information Theory

J. Ricardo Arias-Gonzalez[1,2,*]

[1]Instituto Madrileño de Estudios Avanzados en Nanociencia, C/ Faraday 9, Cantoblanco, 28049 Madrid, Spain

[2]CNB-CSIC-IMDEA Nanociencia Associated Unit "Unidad de Nanobiotecnología", Madrid, Spain

[*]E-mail: ricardo.arias@imdea.org

Supplementary material list

This document includes supplementary sections covering the following information:

- Toy model formulation
- Toy model results
  - Information Potentials,
  - Correctness as a function of the number of errors
  - Critical Points in Effective Revision

S.1 Toy Model Formulation

We next formulate the adaptation a toy model that was previously used in the context of DNA replication and transcription [1, 2]. This model is therefore suitable to describe the copy of information in the presence or absence of revision.

The system under study is a stochastic sequence of symbols,

$$\nu = \{x_1, x_2, \ldots, x_i, \ldots, x_{n-1}, x_n\}\,, \tag{S1}$$

for which the memory at each position i is constituted by the $i-1$ previous symbols. Computation takes place by assembling the symbols in the same way as a so-called Turing machine, i.e., by manipulating the symbols $1 \to n$, one at a time, according to fixed rules. The alphabet, $\mathcal{X}$, is conformed by a number $|\mathcal{X}|$ of symbols.

To describe the neighboring interactions that give rise to the memory, we will use simple correctness functions with explicit dependence on the random variables and the memory unit position i: $C\left(x_i; x_{i-1}, \ldots, x_1\right) = f\left(C_1(x_1), \ldots, C_i(x_i); i\right)$, where $C_i(x_i)$ represents the correctness of an individual symbol in the chain at position i when its interactions with its previous neighbors are negligible (independent variables). To address the neighboring-interaction strength, we introduce a real parameter, α, which increases when interactions monotonously become weaker and fulfills that $\alpha \to +\infty$ in the limit of no interactions. We propose then partial correctness functions with linear dependence on the independent correctnesses:

$$C_i\left(x_i; x_{i-1}, \ldots, x_1\right) = \sum_{j=1}^{i} \kappa(i-j;\alpha) C_j(x_j), \tag{S2}$$

where κ is a kernel function appearing in the exponent of the probability distribution, as expressed in Eq. (1) of the main text (see also [1]). This kernel can be positive or negative, addressing positive or negative feedbacks, respectively. To illustrate representative information systems, we will further provide κ with the next properties:

$$\lim_{j\to i} \kappa(i-j;\alpha) = 1, \tag{S3}$$

$$\lim_{\alpha\to+\infty} \kappa(i-j;\alpha) = \delta_{ij}, \tag{S4}$$

The first condition sets the correctness of the current symbol in the absence of previous neighbor interactions and the second one allows to recover the case of independent variables. For simulations, we will use the following power-law kernel function:

$$\kappa(i-j;\alpha) = \begin{cases} 1, & j = i \\ +(-)1/(i-j+1)^{\alpha}, & j < i, \end{cases} \tag{S5}$$

where the + and − signs for $j < i$ address positive and negative feedbacks, respectively. The total correctness of a sequence is (see Eq. (3) in the main text):

$$C_\nu = \sum_{i=1}^{n} \sum_{j=1}^{i} \kappa(i-j;\alpha) C_j(x_j). \tag{S6}$$

This statistical simplicity is sufficient to strengthen the differences between writing and revision statistics.

We propose the next correctness spectrum:

$$C_i(x_i) = \begin{cases} +\xi, & x_i \text{ correct} \\ -\xi, & x_i \text{ error}, \end{cases} \tag{S7}$$

where ξ is a real, positive number. Additionally, we propose that at each position i there is only one so-called correct x_i, being the rest errors. As explained in the main text, if memory effects are sufficiently strong and the feedback negative, the labels *correct* and *error* in Eq. (S7) may be assumed to reverse due to the counter-balancing effect of the previous memory units.

S.2 Toy Model Results

S.2.1 Information Potentials

The following are the potentials for the writing process:

$$\begin{aligned} A^{(w)}(B,\xi,|\mathcal{X}|;\alpha) &= R\log_a Q^{(id)}(B\xi,|\mathcal{X}|) + \xi\Lambda^{(id)}(B\xi,|\mathcal{X}|)\left(\phi(\alpha)-n\right), & \text{(S8)} \\ C^{(w)}(B,\xi,|\mathcal{X}|;\alpha) &= \xi\phi(\alpha)\Lambda^{(id)}(B\xi,|\mathcal{X}|), & \text{(S9)} \\ H^{(w)}(B\xi,|\mathcal{X}|) &= \log_a Q^{(id)}(B\xi,|\mathcal{X}|) - nB\xi\Lambda^{(id)}(B\xi,|\mathcal{X}|). & \text{(S10)} \end{aligned}$$

The following are the potentials for the revision (proofreading + editing) process:

$$\begin{aligned} A^{(r)}(B,\xi,|\mathcal{X}|;\alpha) &= R\log_a Q(B\xi,|\mathcal{X}|;\alpha), & \text{(S11)} \\ C^{(r)}(B,\xi,|\mathcal{X}|;\alpha) &= \xi\sum_{i=1}^{n}\varphi_i(\alpha)\Lambda_i(B\xi,|\mathcal{X}|;\alpha), & \text{(S12)} \\ H^{(r)}(B\xi,|\mathcal{X}|;\alpha) &= \log_a Q(B\xi,|\mathcal{X}|;\alpha) - B\xi\sum_{i=1}^{n}\varphi_i(\alpha)\Lambda_i(B\xi,|\mathcal{X}|;\alpha). & \text{(S13)} \end{aligned}$$

Finally, the following are the potentials for the case of independent variables (id), describing the information chain in the absence of memory ($\alpha \to +\infty$). In these conditions, the

potentials are equivalent for the writing and the revision processes (see the *Independence Limit* theorem [1]):

$$
\begin{aligned}
A^{(id)}(B,\xi,|\mathcal{X}|) &= R\log_a Q^{(id)}(B\xi,|\mathcal{X}|), && \text{(S14)}\\
C^{(id)}(B,\xi,|\mathcal{X}|) &= n\xi\Lambda^{(id)}(B\xi,|\mathcal{X}|), && \text{(S15)}\\
H^{(id)}(B\xi,|\mathcal{X}|) &= H^{(w)}(B\xi,|\mathcal{X}|). && \text{(S16)}
\end{aligned}
$$

Factors Λ_i and $\Lambda^{(id)}$ are:

$$
\begin{aligned}
\Lambda_i(B\xi,|\mathcal{X}|;\alpha) &= \frac{a^{B\xi\varphi_i(\alpha)}-(|\mathcal{X}|-1)a^{-B\xi\varphi_i(\alpha)}}{a^{B\xi\varphi_i(\alpha)}+(|\mathcal{X}|-1)a^{-B\xi\varphi_i(\alpha)}}, && \text{(S17)}\\
\Lambda^{(id)}(B\xi,|\mathcal{X}|) &= \frac{a^{B\xi}-(|\mathcal{X}|-1)a^{-B\xi}}{a^{B\xi}+(|\mathcal{X}|-1)a^{-B\xi}}, && \text{(S18)}
\end{aligned}
$$

and partial and total sums of the kernel, κ, are, respectively:

$$
\begin{aligned}
\varphi_i(\alpha) &= \sum_{j=1}^{i}\kappa(i-j;\alpha), && \text{(S19)}\\
\phi(\alpha) &= \sum_{i=1}^{n}\varphi_i(\alpha). && \text{(S20)}
\end{aligned}
$$

The standard, sequence-dependent and *id* partition functions read:

$$
\begin{aligned}
Q &= \prod_{i=1}^{n}Q'_i = \prod_{i=1}^{n}\left[a^{B\xi\varphi_i(\alpha)}+(|\mathcal{X}|-1)a^{-B\xi\varphi_i(\alpha)}\right], && \text{(S21)}\\
Q_\nu &= \prod_{i=1}^{n}Q_i = \prod_{i=1}^{n}\left[a^{B\xi}+(|\mathcal{X}|-1)a^{-B\xi}\right]\\
&\quad\times\operatorname{axp}\left(B\sum_{j=1}^{i-1}\kappa(i-j;\alpha)C_j(x_j)\right), && \text{(S22)}\\
Q^{(id)} &= \left[a^{B\xi}+(|\mathcal{X}|-1)a^{-B\xi}\right]^n, && \text{(S23)}
\end{aligned}
$$

where $\operatorname{axp}(x)$ stands for a^x. It is important to note that while index i runs over the ordered sequence positions, $1,\ldots,n$, in Eq. (S22), this index is strictly not related to sequence positions in Eq. (S21).

S.2.2 Correctness as a function of the number of errors

The correctness for a sequence ν as a function of the number of errors, m, is:

$$
C_\nu(m) = \xi\left[\phi(\alpha)-2\sum_{h=1}^{m}\left(1+\sum_{i=k_h+1}^{n}\kappa(i-k_h;\alpha)\right)\right]. \qquad \text{(S24)}
$$

where $i = k_h$ indicates the position of error h ($h = 1, \dots, m$). The demonstration of this expression follows induction. $C_\nu(m)$ takes different values depending on where the errors are located and on the existence of degenerate levels.

For the case of non-interacting symbols (independent variables),

$$C_\nu^{(id)}(m) = \xi\,(n - 2m)\,. \tag{S25}$$

It is clear that the *id*-correctness scaled to the number of memory units, $C_\nu^{(id)}/n$, only depends on m/n (see Fig. 1 in the main text).

S.2.3 Critical Points in Effective Revision

Critical points for $A^{(r)} - A^{(w)}$, $C^{(r)} - C^{(w)}$ and $H^{(r)} - H^{(w)}$ as functions of the stability contrast ξ are given by the next equations, respectively:

$$-\sum_{i=1}^{n} \varphi_i \Lambda_i + \phi \Lambda^{(id)} = (\ln a) B\xi \left(1 - {\Lambda^{(id)}}^2\right)(n - \phi)\,, \tag{S26}$$

$$-\sum_{i=1}^{n} \varphi_i \Lambda_i + \phi \Lambda^{(id)} = (\ln a) B\xi \left[\sum_{i=1}^{n} \varphi_i^2 \left(1 - \Lambda_i^2\right) - \phi\left(1 - {\Lambda^{(id)}}^2\right)\right], \tag{S27}$$

$$0 = (\ln a) B\xi \left[\sum_{i=1}^{n} \varphi_i^2 \left(1 - \Lambda_i^2\right) - n\left(1 - {\Lambda^{(id)}}^2\right)\right]. \tag{S28}$$

It is straightforward to see that $\xi = 0$ is a critical point for the three potential differences. Other critical points like those near $B\xi = 1$, see Figs. 2, 3 and 4 in the main text, or the asymptotic critical point for $B\xi \to +\infty$ may appear depending on the behavior of the memory kernel. Critical points near $B\xi = 1$ appear at different positions in ξ for the three potentials since Eqs. (S26)-(S28) are different.

Supplementary References

[1] Arias-Gonzalez, J. R. Information management in DNA replication modeled by directional, stochastic chains with memory. *J. Chem. Phys.* **145**, 185103 (2016).

[2] Arias-Gonzalez, J. R. Thermodynamic framework for information in nanoscale systems with memory. *J. Chem. Phys.* **147**, 205101 (2017).
